# Cost-effectiveness of targeted therapy versus chemotherapy for patients with advanced or metastatic gastric cancer in China

**DOI:** 10.3389/fphar.2026.1840088

**Published:** 2026-08-10

**Authors:** Kehui Meng, Meiyu Wu, Andong Li, Zixuan Zhang, Yangke Yi, Chaohao Shi, Xiaomin Wan, Chongqing Tan, Hao He

**Affiliations:** 1 Department of Pharmacy, The Second Xiangya Hospital, Central South University, Changsha, Hunan, China; 2 Institute of Clinical Pharmacy, Central South University, Changsha, Hunan, China; 3 Department of Vascular Surgery, The Second Xiangya Hospital of Central South University, Changsha, Hunan, China

**Keywords:** advanced gastric cancer, biomarker testing, China, cost-effectiveness, precision medicine

## Abstract

**Background:**

Multi-biomarker testing increasingly guides first-line therapy for advanced or metastatic gastric cancer in China, but its economic value is uncertain.

**Methods:**

We conducted a Chinese healthcare payer–perspective cost-effectiveness analysis comparing a testing-guided pathway with No-testing, using a hybrid decision tree plus three-state Markov model for a 1,000-patient cohort over 10 years.

**Results:**

Testing-guided care increased costs by $64,242.13 and quality-adjusted life years (QALYs) by 0.63, yielding an incremental cost-effectiveness ratio (ICER) of $101,278.5/QALY and failing to meet the willingness to pay (WTP) threshold. Probabilistic sensitivity analysis (PSA) showed 0% probability of cost-effectiveness at $40,629/QALY; key one-way drivers included progression-free (PF) utility and zolbetuximab cost.

**Conclusions:**

Biomarker-guided treatment improved outcomes but was not cost-effective in China at current prices, mainly due to high drug costs in large biomarker-defined groups. Value-based pricing may improve affordability and cost-effectiveness.

## 1 Introduction

Gastric cancer remains a major global health burden. In 2022, there were 968,784 new cases and 660,175 deaths worldwide, including 358,672 new cases and 260,372 deaths in China (Bray et al., 2024). In China, stomach cancer remains among the leading causes of cancer incidence and mortality, creating substantial pressure on the healthcare system (Han et al., 2024).

Treatment for advanced or metastatic gastric cancer has shifted from empiric chemotherapy toward biomarker-guided targeted therapy and immunotherapy. Evidence supporting HER2-directed therapy, PD-1 inhibitors, and CLDN18.2-directed therapy has expanded first-line treatment options, and current Chinese guidelines increasingly emphasize biomarker assessment to guide treatment selection (Bang et al., 2010; Janjigian et al., 2021; Doki et al., 2022; Shah et al., 2023; Shitara et al., 2023; Janjigian et al., 2024a; Wang et al., 2024; Ajani et al., 2025; Chinese Society of Clinical Oncology (CSCO), 2025).

These advances also bring clear challenges. New regimens are often expensive, raising affordability concerns in China. At the health-system level, access is shaped by price negotiation and reimbursement policies, with a growing role for health technology assessment to support value-based decisions (Zhang et al., 2021; Huang et al., 2022; Liu et al., 2025; Qiu et al., 2025). At the same time, precision care depends on reliable testing, and testing can be difficult in routine practice. Tissue may be limited, results may vary across assays and readers, and tumor heterogeneity can cause discordant findings (Yang et al., 2012; Park et al., 2016; Robert et al., 2023; Fassan et al., 2024; Klempner et al., 2024). These practical issues can delay treatment and reduce confidence in eligibility decisions.

Economic evidence for biomarker-based treatment in gastric cancer is growing, but it is still incomplete. Unlike previous China-based pharmacoeconomic studies that mainly evaluated a single drug, regimen, or biomarker-defined subgroup, this study assessed an integrated multi-biomarker testing-guided pathway in which several clinically actionable biomarkers were linked to first-line treatment allocation within the same model. Many studies evaluate one drug or one biomarker group, so the results do not reflect the full shift toward multi-biomarker care (Bang et al., 2010; Wang et al., 2024; Ajani et al., 2025). In China, value and access are also shaped by pricing and reimbursement policies, so cost-effectiveness results can change with drug prices and coverage decisions (Zhang et al., 2021; Huang et al., 2022; Liu et al., 2025; Qiu et al., 2025). A clear, China-focused economic assessment is needed to support fair access and efficient use of high-cost targeted and immune therapies (Zhang et al., 2021; Huang et al., 2022; Shah et al., 2023; Liu et al., 2025; Qiu et al., 2025). Therefore, this study evaluates the cost-effectiveness of a testing-guided treatment approach versus empiric chemotherapy for first-line advanced gastric cancer in China.

## 2 Methods

We conducted a model-based cost-effectiveness analysis from the Chinese healthcare payer perspective comparing a testing-guided strategy with no-testing for advanced or metastatic gastric cancer. Outcomes were projected over 10 years with 3-week cycles and 5% annual discounting; costs were converted using 1 USD = 7.07 CNY and evaluated against a WTP threshold of 3× China’s *per capita* GDP ($40,629/QALY) (Liu et al., 2020; National Bureau of Statistics, 2025). The model was built in R (version 4.5.2).

### 2.1 Model structure and treatment strategies

**FIGURE 1:**
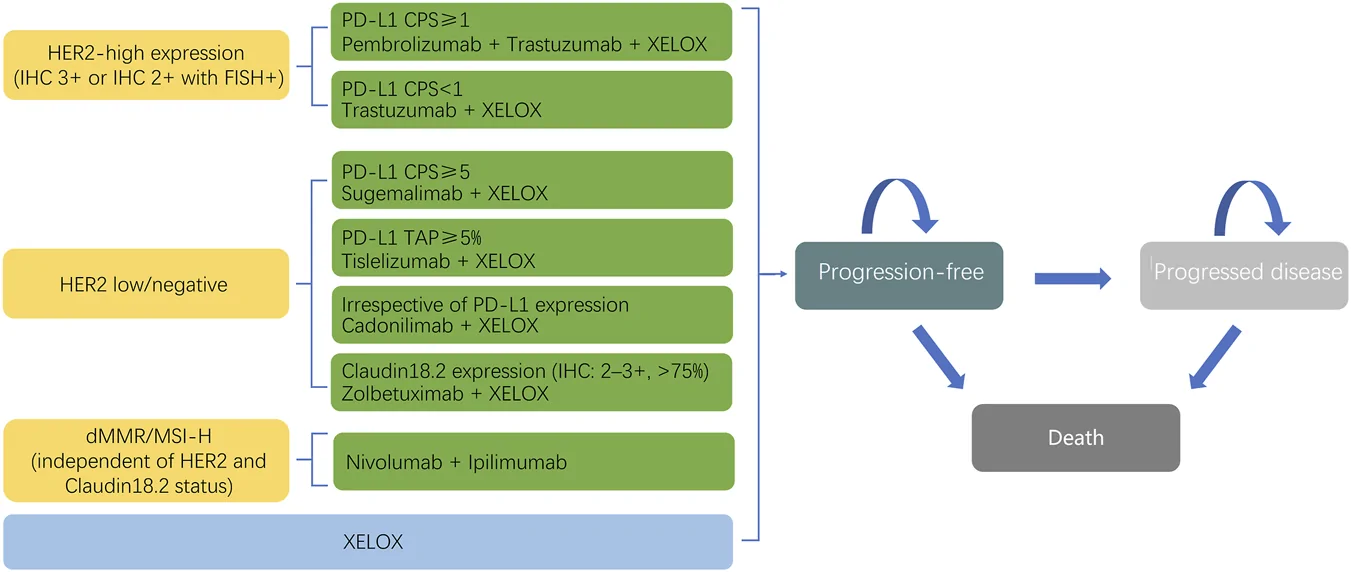
Treatment pathways and model structure.

We used a hybrid model that combined a decision tree and a three-state Markov model. The full pathway is shown in Figure 1. The model simulated a hypothetical cohort of 1,000 patients with advanced or metastatic gastric cancer. In the Testing-guided strategy, patients were distributed across biomarker-defined branches in the decision tree according to the prevalence of each biomarker, using a hierarchical and mutually exclusive algorithm: deficient mismatch repair (dMMR)/microsatellite instability–high (MSI-H) first, then HER2; for HER2-positive tumors, programmed death-ligand 1 (PD-L1) combined positive score (CPS) guided treatment selection; for HER2-negative tumors, CLDN18.2 was prioritized before PD-L1, followed by PD-L1 CPS ≥5 and PD-L1 tumor area positivity (TAP) ≥5% branches, which were assigned to sugemalimab plus XELOX and tislelizumab plus XELOX, respectively; patients negative for all modeled biomarkers received cadonilimab plus XELOX. This hierarchy was used to construct mutually exclusive treatment branches and was based on current Chinese guideline recommendations and available first-line evidence, rather than indicating a biological priority among biomarkers (Chinese Society of Clinical Oncology (CSCO), 2025). In the base-case analysis, we modeled false negatives but assumed no false positives, because positive results for clinically actionable biomarkers are generally subject to pathological review, confirmatory testing, and clinical interpretation before treatment decisions. This assumption was adopted to focus on the loss of treatment opportunity caused by false-negative results.

After progression, both strategies used paclitaxel as subsequent therapy (Wang et al., 2024). Paclitaxel was used as a common subsequent therapy assumption to reduce heterogeneity from diverse post-progression treatment sequences and to focus the comparison on the economic impact of first-line biomarker-guided treatment allocation. Testing was mainly immunohistochemistry (IHC), with reflex *in situ* hybridization (ISH) for HER2 IHC 2+, and regimen details are summarized in Table 1. The Markov model included PF, progressed disease (PD), and death; patients started in PF and could transition to PD or death over time. A three-state structure was selected because the main objective was to compare first-line treatment allocation under different testing strategies, and the key clinical outcomes were captured through regimen-specific PFS and OS estimates. Additional tunnel states or treatment-specific health states were not included to avoid over-parameterization, as treatment differences were already reflected in the survival functions used to derive transition probabilities.

**TABLE 1:** Treatment regimens included in the model.

Group	Treatment regimens	Source
HER2-high, PD-L1 CPS ≥1	Pembrolizumab is administered at 200 mg every 3 weeks (Q3W), in combination with trastuzumab (8 mg/kg loading dose followed by 6 mg/kg Q3W) plus XELOX.	Janjigian et al. (2024b)
HER2-high, PD-L1 CPS <1	Trastuzumab is administered at an 8 mg/kg loading dose followed by 6 mg/kg Q3W, in combination with XELOX.	Bang et al. (2010)
HER2-low/negative, PD-L1 CPS ≥5	Sugemalimab is administered at 1,200 mg every 21 days for up to 24 months, in combination with XELOX.	Zhang et al. (2025a)
HER2-low/negative, PD-L1 TAP ≥5%	Tislelizumab is administered at 200 mg Q3W for up to 2 years, in combination with XELOX.	Qiu et al. (2024)
All biomarkers negative	Cadonilimab is administered at 10 mg/kg Q3W, in combination with XELOX.	Shen et al. (2025)
HER2-low/negative, Claudin18.2-positive	Zolbetuximab is administered at an 800 mg/m^2^ loading dose followed by 600 mg/m^2^ every 21 days, in combination with XELOX.	Shah et al. (2023)
dMMR/MSI-H	Nivolumab is administered at 240 mg every 2 weeks, combined with ipilimumab at 1 mg/kg every 6 weeks, for a maximum duration of 24 months.	Kawakami et al. (2025)
No testing	XELOX	Shen et al. (2025)
Second-line	Paclitaxel is administered at a dose of 200 mg/m^2^ intravenously on day 1 of each 21-day cycle.	(Chinese Society of Clinical Oncology (CSCO), 2025)

CPS, combined positive score; TAP, tumor area positivity; XELOX, capecitabine plus oxaliplatin; Q3W, every 3 weeks.

### 2.2 Model progression and survival estimates

We estimated transition probabilities mainly from trial-based PFS and OS data. We extracted KM data points using WebPlotDigitizer. We reconstructed individual patient-level data using IPDfromKM version 0.1.10. We fitted several parametric models, including Exponential, Weibull, Lognormal, Log-logistic, and Gompertz. We selected the base-case model using Akaike information criterion (AIC). We then calculated cycle-to-cycle transition probabilities using standard methods:

$$t_{p}\left(t_{u}\right)=1‐S\left(t\right)/S\left(t‐u\right)$$

The results of AIC for each parametric model were represented in Supplementary Tables S1 and S2. We performed model validation, and the results showed that the model estimates for PFS and OS for each treatment regimen were consistent with published study findings (Supplementary Figures S1 and S2). We also included age-specific mortality from other causes. These rates were taken from the China Population Census Yearbook 2020 (Office of the Leading Group of the State Council for the Seventh National Population Census, 2023).

### 2.3 Utilities, costs, and adverse events

We assigned health-state utilities for PF and PD. We included disutility and management costs for grade 3 or higher adverse events (AEs), based on clinically important events for each regimen. We included drug acquisition and administration, routine follow-up, biomarker testing, imaging and laboratory tests, and AE management costs. These costs represent direct medical costs borne by the healthcare payer. All model inputs were obtained from published literature or government sources. Drug prices were sourced from the Yaozhi drug database (Yaozhi, 2025). We converted nivolumab costs to a 3-week equivalent to match the cycle. This adjustment was applied to costs only, and state transitions were still simulated in 3-week cycles. All key effectiveness and cost parameters, as well as ranges and distributions for sensitivity analysis, are reported in Table 2.

**TABLE 2:** Model parameters, base-case values, ranges, distributions, and sources.

Parameter	Base case	Range	Distribution	Source
Cost input, US$				
Pembrolizumab cost	25.34/mg	20.27–30.41	Gamma	Yaozhi (2025)
Trastuzumab cost	1.68/mg	1.34–2.02	Gamma	Yaozhi (2025)
Sugemalimab cost	2.92/mg	2.34–3.50	Gamma	Yaozhi (2025)
Tislelizumab cost	1.77/mg	1.42–2.12	Gamma	Yaozhi (2025)
Cadonilimab cost	6.98/mg	5.58–8.38	Gamma	Yaozhi (2025)
Zolbetuximab cost	4.95/mg	3.96–5.94	Gamma	Yaozhi (2025)
Nivolumab cost	13.61/mg	10.89–16.33	Gamma	Yaozhi (2025)
Oxaliplatin cost	0.24/mg	0.19–0.29	Gamma	Yaozhi (2025)
Capecitabine cost	0.01/mg	0.01–0.01	Gamma	Yaozhi (2025)
Paclitaxel cost	0.32/mg	0.26–0.38	Gamma	Yaozhi (2025)
Terminal care	1,463.22	1,170.58–1755.86	Gamma	Zhang et al. (2025b)
Best supportive care	937.74	750.19–1,125.29	Gamma	Lai et al. (2024)
CT per unit	108.31	86.65–129.97	Gamma	Zhou et al. (2025)
MRI per unit	143.90	115.12–172.68	Gamma	Zhou et al. (2025)
Gastroscopy	46.40	37.12–55.68	Gamma	Jiang et al. (2022)
IHC cost	9.90	7.92–11.88	Gamma	Hunan Healthcare Security Administration (2024)
ISH cost	106.08	84.86–127.30	Gamma	Hunan Healthcare Security Administration (2024)
AE management costs, US$				
Anemia	138.75	111.00–166.50	Gamma	Lai et al. (2024)
Decreased neutrophil count	115.01	92.01–138.01	Gamma	Lai et al. (2024)
Decreased platelet count	1,505.92	1,204.74–1807.10	Gamma	Lai et al. (2024)
Diarrhea	132.87	106.30–159.44	Gamma	Lai et al. (2024)
Neutropenia	657.16	525.73–788.59	Gamma	Zhu et al. (2025)
Vomiting	118.77	95.02–142.52	Gamma	Lai et al. (2024)
Health-state utilities				
PFS	0.797	0.64–0.96	Beta	(Zhang et al., 2025b; Zhu et al., 2025)
PD	0.577	0.46–0.69	Beta	(Zhang et al., 2025b; Zhu et al., 2025)
AE disutilities				
Anemia	0.073	0.06–0.09	Beta	Lai et al. (2024)
Decreased neutrophil count	0.150	0.12–0.18	Beta	Lai et al. (2024)
Decreased platelet count	0.090	0.07–0.11	Beta	Lai et al. (2024)
Diarrhea	0.047	0.04–0.06	Beta	Lai et al. (2024)
Neutropenia	0.090	0.07–0.11	Beta	Lai et al. (2024)
Vomiting	0.103	0.08–0.12	Beta	Lai et al. (2024)
Biomarker positivity				
HER2	0.10	0.08–0.12	Beta	Jing et al. (2023)
PD-L1 CPS ≥1	0.40	0.32–0.48	Beta	Jing et al. (2023)
PD-L1 CPS ≥5	0.17	0.14–0.20	Beta	Mao et al. (2022)
PD-L1 TAP ≥5	0.55	0.44–0.66	Beta	Cruz-Correa et al. (2025)
Claudin18.2	0.40	0.32–0.48	Beta	Mao et al. (2022)
dMMR/MSI-H	0.06	0.05–0.07	Beta	Latham et al. (2019)
Test sensitivity				
HER2	0.93	0.74–1.00	Beta	Xue et al. (2024)
dMMR/MSI-H	0.95	0.76–1.00	Beta	Park et al. (2023)
Claudin18.2	0.77	0.62–0.92	Beta	Pak et al. (2025)
PD-L1	0.80	0.51–0.96	Beta	Park et al. (2020)
Other model settings				
Body surface area, m^2^	1.62	1.30–1.94	Normal	Li et al. (2020)
Weight, kg	65.00	52.00–78.00	Normal	Li et al. (2020)
Discount rate	0.05	0.00–0.08	-	Liu et al. (2020)

### 2.4 Uncertainty analysis

We performed one-way sensitivity analyses (OWSA) and probabilistic sensitivity analyses (PSA) to evaluate the robustness of the model. The OWSA examined the impact of individual parameters on the ICER by varying each across its plausible range. Specifically, parameters were varied by ±20% of base-case values (Table 2), with probabilities capped at 1 when needed, and the discount rate was tested from 0.00 to 0.08. In the PSA, 5,000 Monte Carlo simulations were run by sampling parameter values from their respective distributions. Utility and probability parameters were assigned a beta distribution, and cost parameters were assigned a gamma distribution.

## 3 Results

### 3.1 Base-case analysis

In the base case, the Testing-guided strategy increased costs and QALYs compared with the No-testing strategy. The incremental cost was $64,242.13 and the incremental gain was 0.63 QALYs, which resulted in an ICER of $101,278.5 per QALY. Full results are reported in Table 3. At a willingness-to-pay threshold of $40,629 per QALY, the Testing-guided strategy was not cost-effective.

**TABLE 3:** Base-case results.

Results	Testing-guided strategy	No-testing strategy	Incremental
LYs	2.09	1.22	0.87
QALYs	1.44	0.81	0.63
Total costs, US$	88,504.75	24,262.62	64,242.13
ICER, US$/LY	​	​	73,723.70
ICER, US$/QALY	​	​	101,278.50

ICERs were calculated using unrounded incremental costs and QALYs; therefore, displayed values may not exactly reproduce the ICERs because of rounding.

### 3.2 One-way sensitivity analysis

**FIGURE 2:**
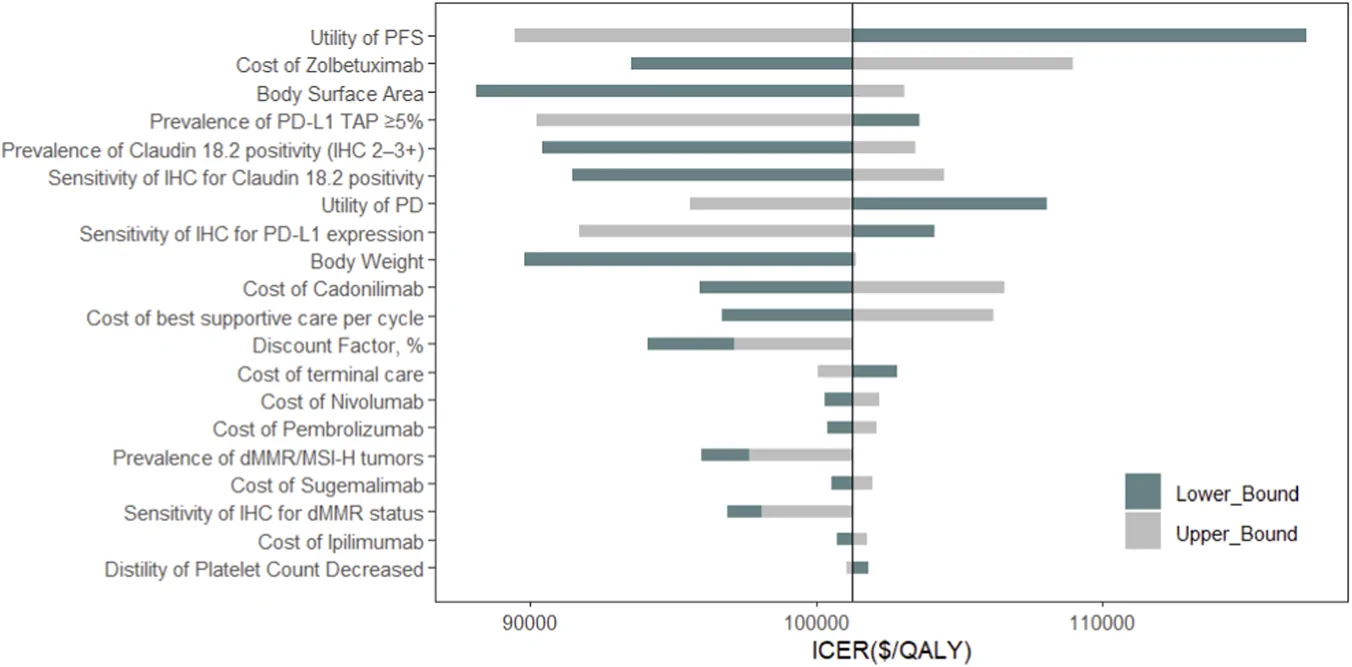
Tornado diagram of one-way sensitivity analysis (ICER outcome) for the Testing-guided strategy versus the No-testing strategy; only the top 20 parameters are shown.

OWSA showed that the ICER was most sensitive to parameters related to health utility and key drug costs. The top drivers included the PF utility, the cost of zolbetuximab, and body surface area. Results for the 20 most influential parameters are shown in the tornado diagram (Figure 2).

### 3.3 Probabilistic sensitivity analysis

**FIGURE 3:**
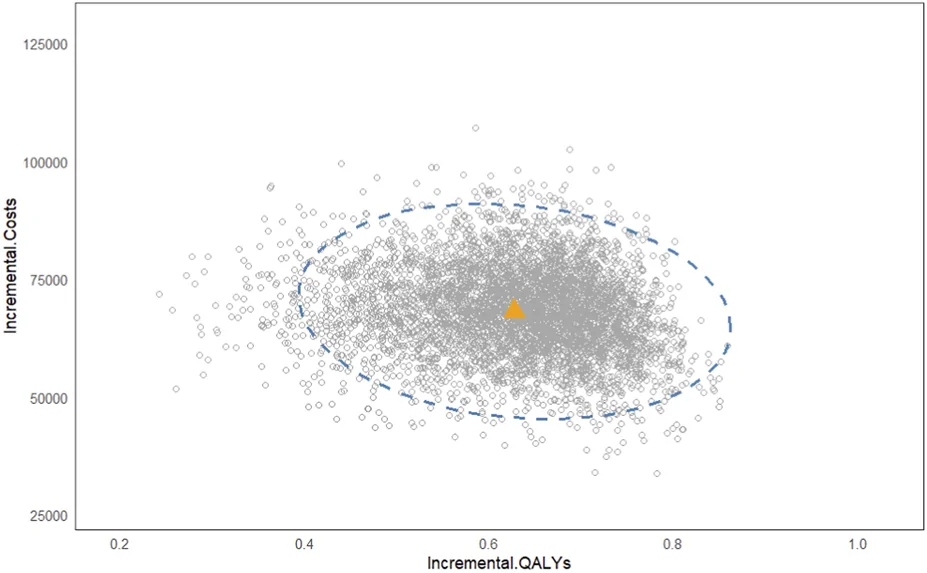
Cost-effectiveness plane from PSA (5,000 iterations) showing incremental costs and QALYs for the Testing-guided strategy versus the No-testing strategy.

**FIGURE 4:**
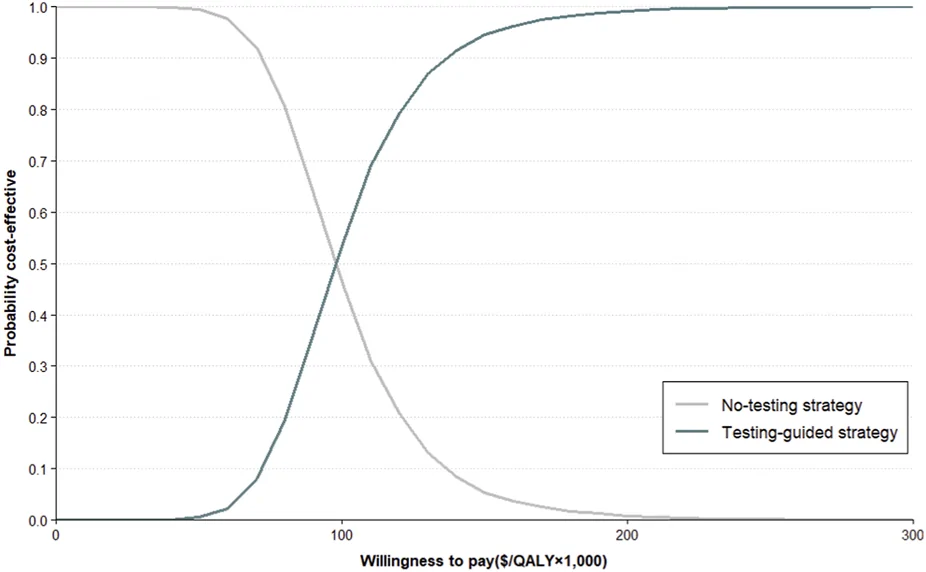
Cost-effectiveness acceptability curve showing the probability that the Testing-guided strategy is cost-effective versus the No-testing strategy across WTP thresholds.

In PSA, the Testing-guided strategy remained more costly than the No-testing strategy across simulations. At the WTP threshold of $40,629 per QALY, the probability that the Testing-guided strategy was cost-effective was 0%. The joint distribution of incremental costs and QALYs is shown in the cost-effectiveness plane (Figure 3), and the CEAC summarizes cost-effectiveness across WTP thresholds (Figure 4).

## 4 Discussion

This study evaluated the cost-effectiveness of a Testing-guided strategy versus the No-testing strategy for advanced or metastatic gastric cancer in China using a hybrid decision tree and Markov model. The Testing-guided strategy increased costs by $64,242.13 and improved outcomes by 0.63 QALYs, resulting in an ICER of $101,278.5 per QALY, which exceeded the prespecified WTP threshold and was therefore not cost-effective.

Our conclusion aligns with many published economic evaluations in gastric cancer showing that modern immunotherapy or targeted combinations are often not cost-effective at current prices under commonly used thresholds. For example, Chinese analyses have reported that nivolumab plus chemotherapy and pembrolizumab-based regimens were not cost-effective compared with chemotherapy-based options unless prices were reduced (Shu et al., 2022). However, our study differs from previous evaluations in scope. This integrated framework represents a key methodological distinction from prior China-based economic evaluations, because it evaluates the testing-to-treatment decision process rather than the cost-effectiveness of an isolated targeted or immune regimen. Many studies focus on one drug or one biomarker-defined subgroup, whereas our analysis modeled a multi-biomarker testing pathway and linked several biomarkers to treatment choices within one framework, which better reflects how treatment selection is increasingly made in practice.

This finding should not be interpreted as evidence against biomarker testing or precision therapy. Targeted therapy and immunotherapy can extend survival and may offer a more favorable toxicity profile for selected patients. The Testing-guided strategy also generated more QALYs in our long-term model. The main driver of the unfavorable ICER was the high acquisition cost of targeted and immune agents, especially drugs used in relatively large biomarker-defined populations. This supports a policy-relevant implication: value-based pricing and reimbursement negotiation are central levers to improve affordability and cost-effectiveness. China has demonstrated the feasibility of large price reductions through national negotiation mechanisms, with studies reporting substantial post-negotiation cost reductions across anticancer indications (Zhou et al., 2024; Xie et al., 2025). Price reductions for the main cost drivers in the testing pathway would be the most direct way to move the ICER toward the WTP threshold.

Sensitivity analyses further identified the parameters that most influenced the ICER. In the OWSA, the price of zolbetuximab was among the strongest drivers of the ICER, partly because the eligible population can be sizeable. CLDN18.2 positivity in gastric cancer has been commonly reported in a substantial proportion of patients, meaning that many patients in the simulated cohort could enter this branch and accumulate high drug costs (Mao et al., 2022). This pattern is consistent with prior economic work specifically evaluating zolbetuximab, where the drug cost and the size of the treated population strongly shaped results and PSA showed a very low probability of cost-effectiveness at Chinese thresholds (Lai et al., 2024). From a payer perspective, these findings suggest that high-impact drugs used by larger biomarker-defined groups may be reasonable candidates for earlier negotiation, price–volume arrangements, or other risk-sharing mechanisms to improve affordability while maintaining access.

These findings also underscore the continuing clinical importance of biomarker testing. Under current prices and assumptions, the Testing-guided strategy was not cost-effective, but testing remains essential for precision care. Biomarker testing can identify patients more likely to benefit from targeted or immune-based therapy and can help avoid exposing biomarker-negative patients to treatments that are unlikely to help and may still cause toxicity. Reviews on CLDN18.2 testing also highlight practical issues in implementation and the need for standardized assays and interpretation, which will be important as testing becomes more routine (Angerilli et al., 2024).

Several limitations should be noted. First, we relied on parametric survival extrapolation from published trials, so long-term outcomes beyond follow-up remain uncertain. Second, biomarker prevalences and test performance were sourced from published evidence and may vary across settings, which could change branch sizes and overall costs. We did not explicitly model false-positive test results, which may underestimate the uncertainty related to inappropriate treatment allocation caused by imperfect test specificity. Future studies with more complete real-world test-performance data should jointly incorporate both false-negative and false-positive results. Third, we used a simplified and uniform subsequent-therapy assumption and did not capture all treatment sequencing patterns, which may affect total costs and QALYs. In real-world practice, post-progression treatment may vary according to patient condition, prior therapy, drug accessibility, and physician preference; therefore, this simplification may influence total costs and QALYs.

Looking forward, improvements in testing workflows, better biomarkers that sharpen patient selection, and lower drug prices could meaningfully improve the cost-effectiveness of testing-guided care.

## Data Availability

The original contributions presented in the study are included in the article/Supplementary Material, further inquiries can be directed to the corresponding authors.
