# Supplementary material for: Cost-effectiveness of targeted therapy versus chemotherapy for patients with advanced or metastatic gastric cancer in China
Source: Front Pharmacol. 2026 Aug 10;17:1840088. doi: 10.3389/fphar.2026.1840088 (PMC13500755; doi:10.3389/fphar.2026.1840088)
Supplement: Supplementary file 1 [file Supplementaryfile1.docx]

Supplementary Material

**Supplementary Material**

**1.Supplementary Figures**

**Figure S1. Parametric fitting of PFS curves.** Panels A–H show the observed Kaplan–Meier PFS curves and the fitted parametric survival functions for each regimen: (A) pembrolizumab plus trastuzumab plus XELOX, (B) trastuzumab plus XELOX, (C) sugemalimab plus XELOX, (D) tislelizumab plus XELOX, (E) cadonilimab plus XELOX, (F) zolbetuximab plus XELOX, (G) nivolumab plus ipilimumab, and (H) XELOX.

**Figure S2. Parametric fitting of OS curves.** Panels A–H show the observed Kaplan–Meier OS curves and the fitted parametric survival functions for each regimen: (A) pembrolizumab plus trastuzumab plus XELOX, (B) trastuzumab plus XELOX, (C) sugemalimab plus XELOX, (D) tislelizumab plus XELOX, (E) cadonilimab plus XELOX, (F) zolbetuximab plus XELOX, (G) nivolumab plus ipilimumab, and (H) XELOX.

**2. Supplementary Tables**

**Table S1.** AIC values for parametric PFS model fits by regimen and distribution.

| Regimen | Exponential | Weibull | Lognormal | Log-logistic | Gompertz | Best-fitting distribution |
| --- | --- | --- | --- | --- | --- | --- |
| Pembrolizumab + trastuzumab + XELOX | 1762.979 | 1764.124 | 1705.802 | 1707.463 | 1734.525 | Lognormal |
| Trastuzumab + XELOX | 1405.550 | 1371.417 | 1336.379 | 1336.715 | 1402.809 | Lognormal |
| Sugemalimab + XELOX | 1211.969 | 1195.094 | 1174.966 | 1167.353 | 1212.835 | Log-logistic |
| Tislelizumab + XELOX | 1194.194 | 1189.141 | 1161.790 | 1159.136 | 1195.372 | Log-logistic |
| Cadonilimab + XELOX | 1257.366 | 1241.590 | 1219.663 | 1222.964 | 1256.518 | Lognormal |
| Zolbetuximab + XELOX | 982.8115 | 966.8504 | 955.3269 | 951.3207 | 981.4093 | Log-logistic |
| Nivolumab + Ipilimumab | 78.68133 | 80.42914 | 79.89556 | 80.41175 | 80.53894 | Exponential |
| XELOX | 1468.515 | 1419.738 | 1391.849 | 1394.211 | 1456.027 | Lognormal |

**Table S2.** AIC values for parametric OS model fits by regimen and distribution.

| Regimen | Exponential | Weibull | Lognormal | Log-logistic | Gompertz | Best-fitting distribution |
| --- | --- | --- | --- | --- | --- | --- |
| Pembrolizumab + trastuzumab + XELOX | 1987.814 | 1984.520 | 1953.377 | 1954.323 | 1988.756 | Lognormal |
| Trastuzumab + XELOX | 1328.849 | 1301.852 | 1307.989 | 1303.044 | 1313.344 | Weibull |
| Sugemalimab + XELOX | 1525.365 | 1522.607 | 1482.817 | 1472.997 | 1524.240 | Log-logistic |
| Tislelizumab + XELOX | 1101.236 | 1090.736 | 1089.262 | 1083.720 | 1100.159 | Log-logistic |
| Cadonilimab + XELOX | 1329.808 | 1312.341 | 1303.486 | 1305.387 | 1324.289 | Lognormal |
| Zolbetuximab + XELOX | 1141.803 | 1117.246 | 1121.761 | 1118.048 | 1127.052 | Weibull |
| Nivolumab + Ipilimumab | 60.98850 | 62.97964 | 62.56260 | 62.89446 | 62.97005 | Exponential |
| XELOX | 1554.155 | 1515.734 | 1540.916 | 1515.908 | 1529.556 | Weibull |
